# The Impact of Intermittent Fasting on Brain-Derived Neurotrophic Factor, Neurotrophin 3, and Rat Behavior in a Rat Model of Type 2 Diabetes Mellitus

**DOI:** 10.3390/brainsci11020242

**Published:** 2021-02-15

**Authors:** Basem H. Elesawy, Bassem M. Raafat, Aya Al Muqbali, Amr M. Abbas, Hussein F. Sakr

**Affiliations:** 1 Department of Clinical Laboratory Sciences, College of Applied Medical Sciences, Taif University, P.O. Box 11099, Taif 21944, Saudi Arabia; b.elesawy@tu.edu.sa; 2 Radiological Sciences Department, College of Applied Medical Sciences, Taif University, P.O. Box 11099, Taif 21944, Saudi Arabia; bassemraafat@hotmail.com; 3 Department of Physiology, College of Medicine and Health Sciences, Sultan Qaboos University, P.O. Box 35, Al Koudh, Muscat PC 123, Oman; s114574@student.squ.edu.om; 4 Department of Physiology, College of Medicine, King Khalid University, Abha 61421, Saudi Arabia; amroabbas1971@gmail.com; 5 Medical Physiology Department, Faculty of Medicine, Mansoura University, Mansoura 35516, Egypt

**Keywords:** intermittent fasting, BDNF, neurotrophin 3, behavior

## Abstract

Type 2 diabetes mellitus (T2DM) is known to be associated with an increased risk of dementia, specifically Alzheimer’s disease and vascular dementia. Intermittent fasting (IF) has been proposed to produce neuroprotective effects through the activation of several signaling pathways. In this study, we investigated the effect of IF on rat behavior in type 2 diabetic rats. Forty male Wistar Kyoto rats were divided into four groups (*n* = 10 for each): the ad libitum (Ad) group, the intermittent fasting group (IF), the streptozotocin-induced diabetic 2 group (T2DM) fed a high-fat diet for 4 weeks followed by a single intraperitoneal (i.p.) injection of streptozotocin (STZ) 25 mg kg^−1^, and the diabetic group with intermittent fasting (T2DM+IF). We evaluated the impact of 3 months of IF (16 h of food deprivation daily) on the levels of brain-derived neurotrophic factor (BDNF), neurotrophin 3 (NT3), serotonin, dopamine, and glutamate in the hippocampus, and rat behavior was assessed by the forced swim test and elevated plus maze. IF for 12 weeks significantly increased (*p* < 0.05) the levels of NT3 and BDNF in both control and T2DM rats. Additionally, it increased serotonin, dopamine, and glutamic acid in diabetic rats. Moreover, IF modulated glucose homeostasis parameters, with a significant decrease (*p* < 0.05) in insulin resistance and downregulation of serum corticosterone level. Interestingly, T2DM rats showed a significant increase in anxiety and depression behaviors, which were ameliorated by IF. These findings suggest that IF could produce a potentially protective effect by increasing the levels of BDNF and NT3 in both control and T2DM rats. IF could be considered as an additional therapy for depression, anxiety, and neurodegenerative diseases.

## 1. Introduction

Recent evidence suggests that metabolic syndrome and type 2 diabetes mellitus (T2DM) are associated with changes in cognitive function, as well as memory [1,2]. Metabolic syndrome is a risk factor for Alzheimer’s disease (AD), and while both conditions have multiple pathological features in common, the most significant is insulin resistance [2,3]. T2DM patients usually complain of poor memory, delayed thinking, prolonged reflex time and lack of attention [4,5].

Reduced executive function and memory loss are connected with lower gray matter density and decreased glucose oxidation in different regions of the brain, such as the orbital and prefrontal cortex [6]. A meta-analysis of 24 studies reported that T2DM patients performed worse on neurocognitive testing compared to non-diabetic controls [4]. Impaired insulin signaling is considered the main etiology in the pathogenesis of diabetic cognitive impairment, [7] as well as increased inflammatory and oxidative stress pathways [8]. It has been stated that insulin receptor functioning is reduced in AD patients. Indeed, reduced insulin receptors expression, as well as desensitization or alterations to their intracellular signaling pathways, have been reported. On the other hand, T2DM is comorbid with depression, and it has been reported that depressive states are twice as common in diabetic patients than in the general population. It has been suggested that depression and T2DM share biological origins, such as an abnormal cytokine-mediated inflammatory response, with hyper activation of innate immunity and over stimulationof the hypothalamo-pituitary adrenal (HPA) axis [9].

Intermittent fasting (IF) is a lifestyle of eating within a specific period of time and fasting for the rest of the day. The eating pattern during the fasting period (16–18 h) involves a low amount of intake or the intake of non-caloric food. There are some energy storage organs in the body, such as adipose tissue and the liver, that enable fasting [10]. IF has been shown to increase lifespan, regulate energy metabolism, and reduce the risk of developing different age-related diseases [11]. Moreover, the rate of physical and mental performance increases during fasting due to improvement in metabolic processes (metabolic shift to ketone bodies) and neurotransmission through increased gamma-amino butyric acid GABA and serotonin signaling [12,13]. Previous experiments demonstrated that IF could have a protective role in many neurodegenerative disorders, including Parkinson’s disease (PD) in both rodents and humans [14,15,16].

Neurogenesis is the formation of new neurons in the brain. Normally, the hippocampus is the main site for neurogenesis under the effect of several neurotrophic factors. The most prevalent neurotrophic factor is brain-derived neurotrophic factor (BDNF), which is considered to play an integral role in stimulating the growth of new brain cells and the performance of existing neurons; BDNF is best described as the brain’s growth hormone. Several factors affect the expression of BDNF, such as exercise, sleep, aging, and dietary habits [17]. The process of neurogenesis is controlled by a variety of factors, such as neurotrophic factors, blood glucose level, insulin, lipid profile, corticosterone level, and oxidative stress. BDNF and neurotrophin 3 (NT3) are members of the neurotrophin family that play important roles in the functioning of the central nervous system, such as synaptic plasticity and the maturation, growth, and maintenance of neurons, which have a great effect on cognitive function and emotion [18,19]. IF has been proposed to be neuroprotective against acute brain injuries, such as stroke, and neurodegenerative diseases [20,21,22]. In addition, IF enhances hippocampal neurogenesis and long-term potentiation at hippocampal synapses [23]. Neural progenitor cell production is upregulated with antidepressant treatment and conversely, Hippocampal neurogenesis is reduced in depressed patients [24]. Similarly, animal models of depression result in reduced hippocampal neurogenesis [25] and antidepressants serve to increase cell proliferation and neurogenesis [26]. It has been suggested that the upregulation of neurogenesis is essential to the efficacy of antidepressant drugs and the alleviation of depressive behaviors [27].

We hypothesized that IF could ameliorate the metabolic dysfunction and behavioral changes seen in diabetic rats. So, we investigated the effects of IF on the markers of neurogenesis in a rat model of T2DM.

## 2. Material and Methods

### 2.1. Animals

Forty male Wistar Kyoto rats were used in the present study. Rats were between 12–14 weeks of age and weighed between 300–400 g. All rats were provided from the animal house in Sultan Qaboos University (SQU) where they were kept under controlled conditions (23 ± 1 °C, 12 h light:12 h dark cycle). All experimental procedures were approved by our local committee of Animal Care. Experiments were performed according to the Laboratory Animals Guide published by the US National Institute of Health (Stapleton, NY, USA) (NIH publication no. 85–23, revised 1996).

### 2.2. Experimental Design

The rats were divided into 4 equal groups of 10 rats each. Group I/control group (ad libitum) included normal male Wistar Kyoto rat fed an ad libitum (AL) diet and served as a control. Group II/IF group (18 h/day fasting) included rats that fasted daily for 18 h (14:00–08:00) for 3 months. Group III/streptozotocin-induced diabetic group (T2DM) included rats in which DM was induced by a single intraperitoneal (i.p.) injection of streptozotocin (STZ). Group IV/T2DM + IF included diabetic rats that were exposed to IF for 3 months.

### 2.3. Induction of Diabetes Mellitus

T2DM rats were fed a diet enriched in fat (25% fat, 15% protein, 51% starch and 5% fiber) [28]. After 4 weeks on their respective diets, experimental rats were injected i.p. with streptozotocin (STZ) (25 mg kg^−1^ body weight) [29] and normal rats were injected with vehicle (0.05 mol L^−1^ citric acid, pH 4.5). Both the low dose of streptozotocin and the high-fat diet are essential elements of the model designed to induce type 2 diabetes with insulin resistance. After 30 days, the post-prandial serum glucose and insulin were measured at 2 and 12 h after giving the rat 20% glucose (3 g kg^−1^ body weight). Only rats with increased postprandial glucose (>200 mg dL^−1^) were considered diabetic. The experiments were conducted in accordance with the ethical guidelines for investigations in laboratory animals and were approved by the ethical committee of the College of Medicine, SQU.

### 2.4. Sampling Protocol

#### Blood Samples

At the end of the experimental period, all rats were anesthetized by an i.p dose of sodium pentobarbital (60 mg/kg). Blood samples were collected without anticoagulant, left for 10 min, and then centrifuged for 10 min at 3000 r/min to obtain serum, which was stored at −20 °C until further biochemical analysis for the detection of serum corticosterone, glucose, and insulin levels.

### 2.5. Tissue Samples: Brain Tissue Preparation

The whole brain was quickly removed and washed in 0.9% cold saline. The hippocampus was carefully dissected and homogenized. The homogenates were centrifuged at 10,000× *g* for 15 min at 4 °C, and the supernatant was kept and stored at –80 °C until used for the determination of BDNF, NT3, 5-hydroxytryptamine (5-HT), dopamine, and glutamic acid levels.

### 2.6. Biochemical Investigations

#### Estimation of BDNF, NT3, 5-HT, Dopamine and Glutamic Acid in the Hippocampus

ELISA assay kits were used for the determination of BDNF (catalog number: MBS2700741, MyBioSource, San Diego, CA, USA), NT3 (catalog number: MBS2701228, MyBioSource, USA), 5-HT (catalog number: MBS725497, MyBioSource, USA), dopamine (catalog number: MBS701755, MyBioSource, USA), and glutamic acid (catalog number: MBS2700682, MyBioSource, San Diego, CA, USA) levels in the hippocampus according to the manufacturer’s instructions.

### 2.7. Calculation of Homeostatic Model Assessment (HOMA) Index

The HOMA of IRs was calculated using the following equation: HOMA = fasting glucose (mmol L^−1^) × fasting insulin (mU L^−1^)/22.5. Typically, a HOMA value > 2 is used to identify significant insulin resistance (IR) reduction [30].

### 2.8. Behavioral Tests

#### Elevated Plus Maze

The elevated plus maze (EPM) test consists of two open arms and two close arms and was performed in a sound-proofed room. The test was carried out at the same time for all rats (in the morning). This test took two consecutive days, the first day of which was a training day for the rats for 15 min to familiarize them with the maze. During the second day, the test was started by putting the rat in the center area and allowing them to explore the maze for five minutes. The time spent in both close and open arms, as well the number of entries in each arm, were calculated. After each test 10% of ethanol solution was used to clean up the maze. The basic test was performed as described by Rodgers and Dalvi [31].

### 2.9. Forced Swim Test (FST)

The depressive behavior induced by the forced swim test was evaluated in all animals using a modified method of Porsolt et al. [32,33]. This process comprises of exposing an animal to a situation of inescapable stress, in which the rat is forced to swim. After an initial period of strong swimming action toward the direction of the tank border (latency of the attempt to escape), the rat decreases the intensity of their activities, just producing the essential movements to keep their head out of the water. This is classified as behavioral immobility, indicating a possible state of despair in the animal when it realizes that there is no escape. The rats were positioned individually in a cylindrical tank (100 cm diameter × 60 cm height) whose level of water did not allow the animal to reach the floor, nor arise over the border. The temperature of the water was kept at 25 °C. The animals were submitted to the forced swim test for 15 min (pre-test). After 15 min of forced swimming, each animal was gently dried and then returned to their cages. Twenty-four hours after the pre-test, all the animals were put back inside the tank. The individual behavioral evaluation was accomplished and quantified during 5 min of forced swimming. The behavioral parameters such as as the latency of the attempt of escape (LAE) and behavioral immobility (BI) were quantified in seconds (s). Then, the animals were removed from the water, gently dried, and placed back into their cages.

### 2.10. Statistical Analysis

The data were expressed as mean ± standard deviation (SD). Data were analyzed using SPSS version 20 (SPSS, Inc., Chicago, IL, USA). One-way analysis of variance (ANOVA) was used, followed by Tukey’s post hoc test. Results were considered significant if *p* ≤ 0.05.

## 3. Results

### 3.1. IF Increased the Hippocampal Level of NT3 and BDNF in Control and Diabetic Rats

**Figure 1:**
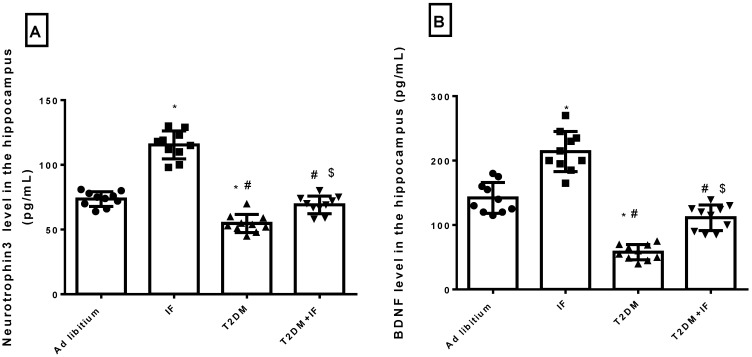
Neurotrophin 3 (NT3) (**A**) and brain-derived neurotrophic factor (BDNF) (**B**) level in the hippocampus in all groups of rats. Data were expressed as mean ± standard deviation (SD) of *n* = 10 rats/group. Values were considered significantly different at *p* < 0.05. *: significantly different as compared to control (ad libitum). #: significantly different as compared to intermittent fasting (IF) group. $: significantly different as compared to type 2 diabetes mellitus (T2DM) group.

Previous studies concluded that IF enhances hippocampal neurogenesis [34] and reduces brain damage by generating new neurons in response to oxidative stress [35]. IF significantly increased (*p* < 0.0001) the level of NT3 and BDNF compared to the control rats. As depicted in Figure 1A,B, the level of NT3 and BDNF in the hippocampus decreased significantly (adjusted *p* < 0.0001) in diabetic rats compared to control rats. Food deprivation for 18 h in T2DM rats significantly increased (*p* < 0.05) NT3 (*p* = 0.0012) and BDNF (adjusted *p* value < 0.0001) levels compared to diabetic rats. The F values for NT3 and BDNF were 37.82 and 31.85, respectively.

### 3.2. IF Modulated the Hippocampal Level of 5-HT, Dopamine and Glutamic Acid in Control and Diabetic Rats

**Figure 2:**
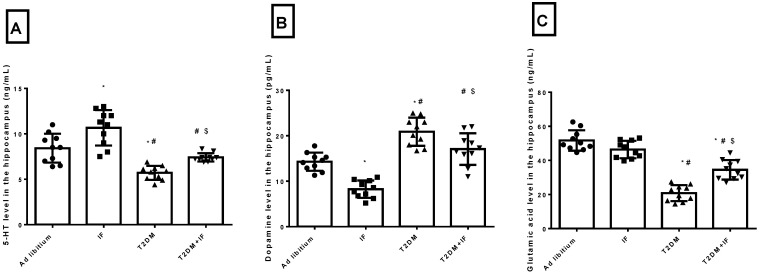
Serotonin (5-HT) (**A**), dopamine (**B**) and glutamic acid (**C**) level in the hippocampus in all groups of rats. Data were expressed as mean ± SD of *n* = 10 rats/group. Values were considered significantly different at *p* < 0.05. *: significantly different as compared to control (ad libitum). #: significantly different as compared to IF group. $: significantly different as compared to T2DM group.

As depicted in Figure 2A–C, IF significantly increased (*p* = 0.0038) the hippocampal level of 5-HT and significantly decreased (*p* < 0.0001) the level of dopamine, with a non-significant (*p* = 0.1426) decrease in glutamic acid compared to ad libitum rats. In T2DM rats, there was a significant decrease in 5-HT (*p* = 0.0004) and glutamic acid (*p* < 0.0001) with a significant increase (*p* < 0.0001) in dopamine compared to the ad libitum rats. IF in diabetic rats increased 5-HT (*p* = 0.0345) and glutamic acid (*p* < 0.0001) significantly with a significant decrease in dopamine (*p* = 0.0164) compared to diabetic rats. Interestingly, T2DM + IF rats showed a non-significant increase (*p* = 0.1183) in dopamine compared to the ad libitum group. The F values for 5-HT, dopamine, and glutamic acid were 33.58, 38.94, and 38.87, respectively.

### 3.3. IF Modulated Glucose Homeostasis Parameters and Corticosterone in Control and Diabetic Rats

**Figure 3:**
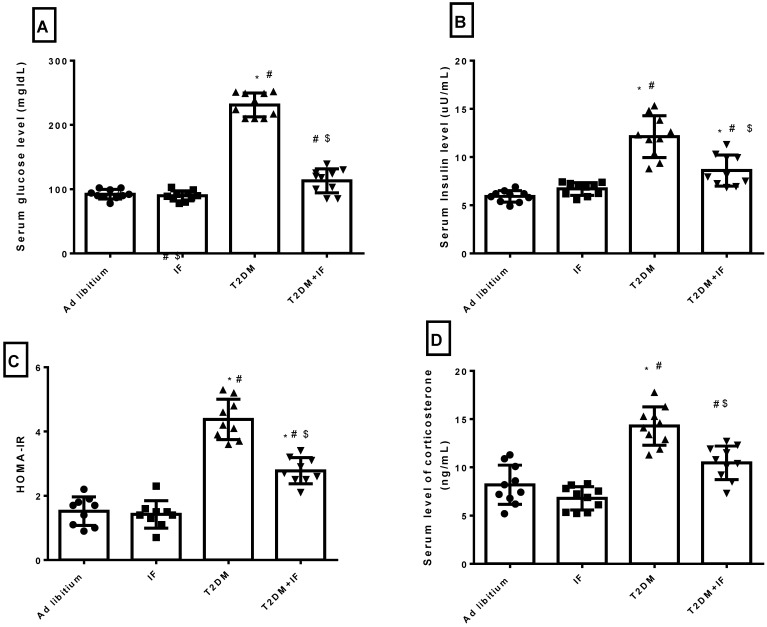
Serum levels of glucose (**A**), insulin (**B**) and Corticosterone (**D**), as well as the calculation of homeostatic model of insulin resistance (HOMA-IR) (**C**) in all groups of rats. Data were expressed as mean ± SD of *n* = 10 rats/group. Values were considered significantly different at *p* < 0.05. *: significantly different as compared to control (ad libitum). #: significantly different as compared to IF group. $: significantly different as compared to T2DM group.

As depicted in Figure 3A–D, IF produced a non-significant decrease in the serum level of glucose (*p* = 0.9791), insulin (*p* = 0.6188), HOMA-IR (*p* = 0.9716) and corticosterone (*p* = 0.3068) compared to the control group. T2DM rats had a significantly increased serum level of glucose (*p* < 0.0001), insulin (*p* < 0.0001), corticosterone (*p* < 0.0001) and HOMA-IR (*p* < 0.0001) compared to the control and IF groups. IF in T2DM rats produced a significant decrease (*p* < 0.0001) in glucose homeostasis parameters compared to diabetic rats. Serum corticosterone decreased significantly (*p* = 0.0002) in T2DM+IF rats compared to the T2DM group.

### 3.4. Effects of IF on the Behavioral Response in the Forced Swim Test

**Figure 4:**
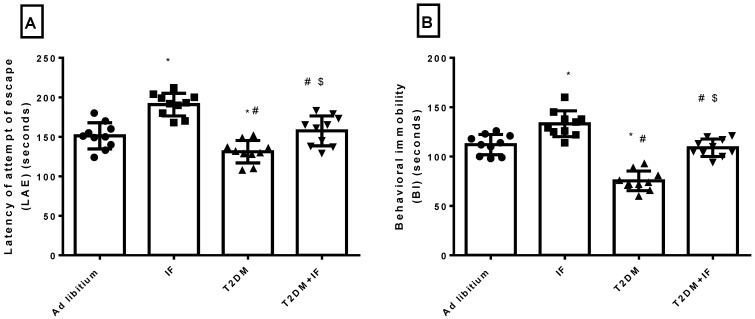
The latency of the attempt of escape (LAE) (**A**) and behavioral immobility (BI) (**B**) in all groups of rats. Data were expressed as mean ± SD of *n* = 10 rats/group. Values were considered significantly different at *p* < 0.05. *: significantly different as compared to control (ad libitum). #: significantly different as compared to IF group. $: significantly different as compared to T2DM group.

As depicted in Figure 4A,B, while LAE was significantly increased (*p* < 0.0001) in animals experiencing IF for 12 weeks compared with the control group (ad libitum), BI was decreased (*p* < 0.0005). T2DM rats showed a significant reduction in both LAE (*p* = 0.0406) and BI (*p* < 0.0001) compared to the ad libitum group. IF in T2DM rats prolonged LAE (*p* = 0.0043) and BI (*p* < 0.0001) compared to the T2DM group. Interestingly, the effects of IF in T2DM rats on LAE (*p* = 0.8190) and BI (*p* = 0.9073) were non-significant when compared to ad libitum rats.

### 3.5. IF Reduced Anxiety in Both Control and Diabetic Rats

**Figure 5:**
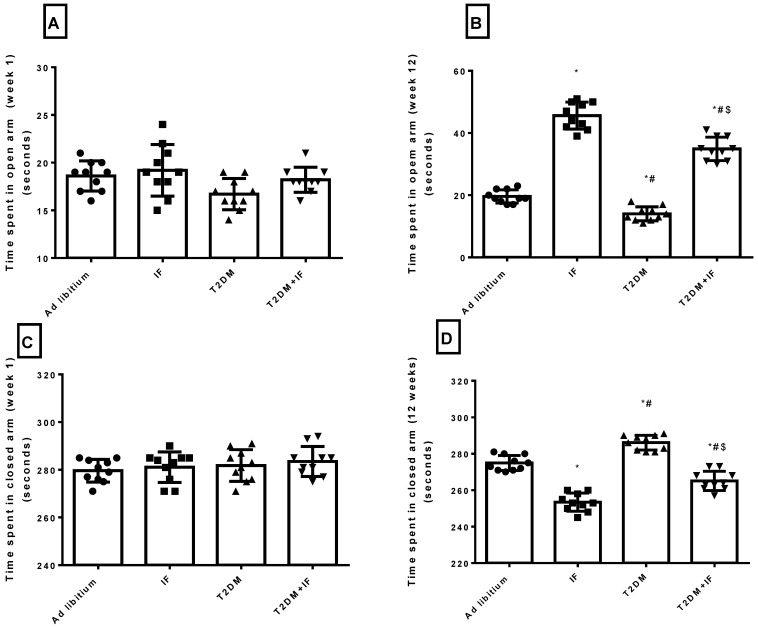
Elevated plus maze results. Time spent in open arm—week 1 (**A**), time spent in open arm—week 12 (**B**), time spent in closed arm—week 1 (**C**) and time spent in closed arm—week 12 (**D**) in all groups of rats. Data were expressed as mean ± SD of *n* = 10 rats/group. Values were considered significantly different at *p* < 0.05. *: significantly different as compared to control (ad libitum). #: significantly different as compared to IF group. $: significantly different as compared to T2DM group.

As depicted in Figure 5A–D, in response to IF for 12 weeks, there was a significant increase in the time spent in the open arm of the elevated plus maze (*p* = 0.0123) compared to the time spent in week 1. Moreover, the time spent in the closed arm decreased significantly (*p* = 0.0174) after 12 weeks of IF compared to the time spent in week 1. The ad libitum group showed a non-significant change in the times spent in both the open and closed arms after 12 weeks. Interestingly, diabetic rats showed a significant decrease (*p* < 0.01) in time spent in the open arm in the first week, with a non-significant change in the time spent in the closed arm as compared to ad libitum rats. After 12 weeks, T2DM rats showed a significant increase (*p* < 0.05) in the time spent in the closed arm with a significant decrease (*p* < 0.01) in the time spent in the open arm compared to their performance in the first week. Additionally, versus ad libitum rats, diabetic rats stayed longer in the closed arm and shorter in the open arm after 12 weeks. T2DM + IF rats showed a significant prolongation (*p* < 0.05) in the time spent in the open arm in week 1 and week 12 compared to T2DM rats. Moreover, T2DM + IF rats reduced the time spent in the closed arm significantly (*p* < 0.05) after 12 weeks compared to the time spent in the first week.

### 3.6. IF Reduced the Final Body Weight and the Percentage of Weight Gain

As shown in Table 1, IF significantly decreased (*p* < 0.05) the final body weight and the percent weight gain at the end of the study compared to control rats. As compared to control rats, T2DM rats showed a significant increase (*p* < 0.01) in body weight, as well as percent weight gain. IF in diabetic rats decreased the body weight significantly (*p* < 0.05) compared to the T2DM group.

### 3.7. Correlations between Different Parameters

**Figure 6:**
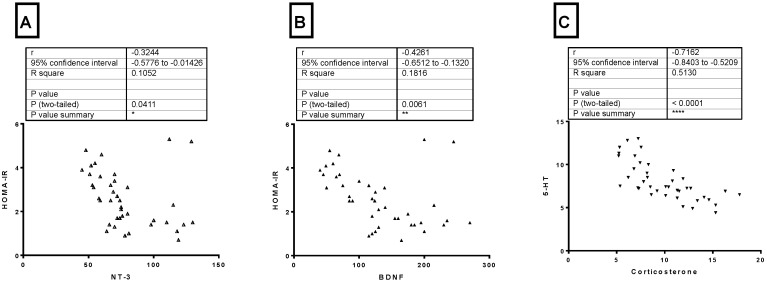
(**A**) Pearson correlation between HOMA-IR and NT3 level in the hippocampus, (**B**) Pearson correlation between HOMA-IR and BDNF level in the hippocampus, and (**C**) Pearson correlation between serum corticosterone and 5-HT level in the hippocampus. Neurotrophin3: NT3 *: *p* < 0.05, **: *p* < 0.01, ****: *p* < 0.0001.

From Figure 6 (A–C), there was a significant negative correlation (*p* = 0.0411) between HOMA-IR and NT3 level in the hippocampus (r = −0.3244). Also, there was a significant negative correlation (*p* = 0.0061) between HOMA-IR and BDNF level in the hippocampus (r = −0.4261). The serum level of corticosterone and 5-HT level in the hippocampus showed a significant negative correlation (*p* < −0.0001) (r = −0.7162).

## 4. Discussion

The main findings of this study include: (1) IF for 12 weeks increases neurogenesis markers such as BDNF and NT3 in both control and diabetic rats; (2) IF reduces anxiety and depression as indicated by elevated plus maze test times and the forced swim test, respectively; (3) IF modulates the expression of neurotransmitters in T2DM rats; and (4) IF ameliorates metabolic dysfunction in T2DM rats.

Reduced formation of new neurons and decreased synaptic plasticity have been implicated in the pathogenesis of different psychological disorders such as anxiety and depression. Stimulating hippocampal neurogenesis through either lifestyle modifications such as regular physical exercise and good sleep, or antidepressants, presents a potential new strategy for treating depression. Interestingly, caloric restriction by 30% supports synaptic plasticity and promotes the survival of newly generated cells in the hippocampus [36]. To the best of our knowledge, we are the first to study the relation between IF and NT3 [37]. The effects of NT3 include the modulation of transmitter release at numerous types of synapses in the periphery and in the adult central nervous system CNS [38]. In addition, NT3 may play a role in the development of tissues other than the nervous system, such as the cardiovascular system. Future research will increase the understanding of the many actions of NT3 on both non-neuronal and neuronal cells [39].

Our data showed a significant increase in BDNF and NT3 in both control and T2DM rats in response to fasting. Previous studies have reported similar effects on BDNF level. In humans, during prolonged fasting in a state known as nutritional ketosis, the plasma level of 3-β-hydroxybutyrate is about five times that of free fatty acids and acetoacetic acid [40]. The brain and other tissues such as skeletal muscle and cardiac muscle utilize ketone bodies in a process termed ketolysis, in which acetoacetic acid and 3-β-hydroxybutyrate are converted into acetoacetyl-CoA and then acetyl-CoA [41]. BNDF gene expression is stimulated by 3-β-hydroxybutyrate, which also increases BDNF protein levels in neurons of the cerebral cortex through the activation of the BDNF gene promoter IV by a mechanism involving the transcription factor NF-κB and the histone acetyltransferase p300 [40]. In addition, 3-β-hydroxybutyrate is considered as an energy source for neurons to sustain their function when glucose levels are lowered, such as during prolonged fasting (more than 12 h) [42]. Ketogenic diets and fasting can protect hippocampal neurons against seizure-induced damage [43,44,45]. Fasting and 3-β-hydroxybutyrate can also counteract disease progression and improve functional outcome in animal models of AD and Parkinson’s disease (PD), stroke, and traumatic brain injury [44,46]. Previous clinical studies suggested that ketone bodies could exert therapeutic benefit by slowing the process of neurodegenerative diseases such as AD and PD [47,48]. In agreement with our results, increased insulin sensitivity stimulates the upregulation of BDNF and increased its level in serum [49]. In hippocampal and cortical neurons, ketone bodies play a vital signaling role by inducing the transcription of BDNF through its suppression of histone deacetylases, enzymes that inhibit BDNF expression.

T2DM rats showed a significant decrease in the neurogenic markers BDNF and NT3. Hyperglycemia and peripheral insulin resistance at the level of the CNS reduced the energy supply to the neurons and neuroglia [50]. Additionally, oxidative stress and inflammation contributed to mitochondrial dysfunction and accelerated apoptosis [51]. A higher glucose concentration in brain tissues and higher insulin resistance are linked with the severity of AD and the expression of its symptoms [52]. Moreover, there is a hypothesis that AD is a “diabetes type 3”. Insulin resistance is related to the production of beta-amyloid deposits in the CNS, as it is originally hydrolyzed by insulin-degrading enzymes [53].

Additionally, IF in both control and diabetic rats modulated the level of 5-HT, dopamine, and glutamic acid. In response to fasting in ad libitum and T2DM rats, 5-HT increased significantly. Previous studies showed that fasting and caloric restriction improve depression symptoms [54]. In agreement with our results, other studies on rodent models showed that fasting boosted the obtainability of brain tryptophan and serotonin [55]. Furthermore, the serotonin reuptake transporter (5-HTT) reuptakes 5-HT from the synaptic cleft to store in the vesicles or to be deactivated by monoamine oxidase (MAO) enzymes. Two weeks of 50% food restriction greatly reduced 5-HTT density in the frontal cortex of rats, resulting in increased levels of 5-HT [56].

In the present study, T2DM rats showed a significant reduction in 5-HT level compared to control rats. In agreement with our data, several articles from 1990 to 2013 that studied and analyzed the relation between diabetes and depressive symptoms reported that neuropathologically, a reduction in brain monoaminergic activity (particularly the serotonin (5-HT) system) due to a chronically persisting diabetic state may lead to mood and behavioral complications that further add to a worse quality of life. The exact cellular mechanism that conclusively explains the development of depression in diabetes is still under investigation. However, one possible mechanism may be the enzymatic alterations in the brain (including decreased levels of the free fraction of L-tryptophan (FFT) and the inhibition of tryptophan-5-hydroxy-lase 2 (TPH 2)) that are responsible for the conversion of FFT to 5-HT. It has been established that DM affects the equilibrium of FFT with other neutral amino acids that play an important role in determining the amount of FFT available for conversion to 5-HT, ultimately leading to a downregulation of 5-HT synthesis [57]. The significant decrease in 5-HT explained the shortening of LAE and BI in T2DM rats. Moreover, an increase in 5-HT improved the symptoms of depression in T2DM rats through the prolongation of LAE and BI in response to IF (Figure 4A,B).

Interestingly, T2DM increased the level of dopamine as compared to ad libitum rats. This was associated with a prolongation of a rat’s tendency to stay in the closed arm of the elevated plus maze test (Figure 5A–D). Hyperglycemia and hyperinsulinemia increase dopamine release [58]. This is due to altered mitochondrial function, aberrant MAO expression, and increased dopamine turnover in the mesolimbic system, and can be reversed by treatment with MAO inhibitors. Thus, brain insulin resistance alters dopamine turnover and induces anxiety and depressive-like behaviors [58]. IF significantly decreased the level of dopamine in the hippocampus in both ad libitum and T2DM rats. In disagreement with our results, some studies showed that fasting during Ramadan has no significant effect on dopamine [59]. Moreover, another study revealed that stress did not change dopamine or acetylcholine release in calorie restricted or control rats (30 months of age) [60]. Additionally, in the present study, T2DM significantly deceased glutamic acid level compared to ad libitum rats, while T2DM+IF decreased it significantly versus T2DM rats. Glutamate, which along with GABA makes up about 90% of the neurotransmitters in the CNS, could be decreased in response to oxidative stress as well as insulin resistance as seen in T2DM rats.

In addition, T2DM rats showed a significant increase in the circulating levels of glucose, insulin, and corticosterone, with a significant decrease in insulin sensitivity as compared to ad libitum rats. Interestingly, in response to intermittent fasting in both control and T2DM rats, metabolic disturbances were ameliorated by decreasing glucose, insulin, and corticosterone, with a significant increase in insulin sensitivity. Previous studies concluded that intermittent fasting leads to a decrease in insulin-like growth factor 1 expression and a consequent reduction in glucose levels [12]. Additionally, IF enhances insulin sensitivity in neurons and ameliorates dysfunction in glucose metabolism. One of the mechanisms through which IF increases insulin sensitivity is the depletion of liver and skeletal muscle glycogen and the metabolic shift to ketosis [12]. Fasting is associated with an elevation in insulin-mediated glucose uptake [61]. It has been postulated that Islamic IF may induce weight loss through various mechanisms, including reduced energy intake [62], a reduction of total body fluids, and changes in the serum levels of leptin, insulin, and corticosterone. In addition, the reduction in body weight after Ramadan diurnal fasting (RDF) could be attributed to the metabolic shift to ketogenesis and fatty acid oxidation in response to fasting [62]. Moreover, an IF-induced increase in BDNF reduced food intake [63]. Selective BDNF infusion into the lateral ventricles of the brain decreased food intake and associated excessive weight gain in rats [64]. As shown in Table 1, IF reduced the weight gain both control and diabetic rats. 

Glucocorticoids repress neurogenesis in the dentate gyrus and reduce both cell proliferation and the density of immature neurons in the adult hippocampus in both male and female rats. However, another interesting finding in this study is that, IF in ad libitum rats produced a non-significant change in corticosterone level. This finding disagrees with previous studies, which revealed that IF increased the corticosterone level. This can be explained by the fact that chronic fasting is considered as a chronic stressor, which could alleviate the stimulation of the HPA axis and modify the serum level of corticosterone. These results disagreed with previous studies [65,66,67]. Caloric restrictions have been shown to activate the HPA axis, increasing the level of glucocorticoids and ameliorating depressive symptoms [68]. Fasting produces antidepressant-like effects in mice, accompanied by an increase in glucocorticoids [69]. In response to IF in T2DM rats, the corticosterone level decreased significantly compared to T2DM rats. The mechanism of corticosterone suppression is not clearly understood. The relation between hyperglycemia in T2DM and the adrenal cortex is unclear. Corticosterone participates in the pathogeneses of diabetes mellitus and β cell dysfunction. It can also exert anti-insulin effects and decrease insulin sensitivity. A mechanism that could explain the relation between diabetes and increased corticosterone is that hyperglycemia induces a stress response, leading to the increased production of corticosterone (as measured by increased adreno-corticotrophic hormone (ACTH) levels); another mechanism could involve the impairment of hydroxysteroid dehydrogenase activity by hyperglycemia, resulting in the decreased metabolism of corticosterone [70]. IF increased the serotonin level in both control and diabetic rats with increased BDNF.

Regarding body weight change and weight gain percentage, IF significantly lowered the weight gain in both control and T2DM rats. In agreement with our results, Wan et al. [71] reported that IF decreased weight gain in rats. Chausse et al. [72] also demonstrated that IF modulated endocrine regulation by changing the HPA, resulting in reduced body weight in rats. Clinical trials have also revealed that alternative day fasting is effective for weight loss and cardio-protection in normal weight and overweight adults [73].

## 5. Conclusions

Our data suggest that chronic fasting for 12 weeks ameliorates depression and anxiety behaviors in T2DM rats by increasing the levels of BDNF and NT3. IF could be considered as a therapeutic tool for known risk factors of behavioral changes, including neuroinflammation, synaptic dysfunction, vascular dysfunction, insulin resistance, and neurogenesis.

## Figures and Tables

**Table 1:** The effect of IF in control and T2DM rats on final body weight and weight gain (%). *: *p* < 0.05 significantly different as compared to control. #: significantly different as compared to IF group. $: significantly different as compared to T2DM group.

	Ad Libitum	IF	T2DM	T2DM+IF
Initial body weight	355.1 ± 8.8	347.4 ± 8.2	342.6 ± 7.4	367.6 ± 9.1
Final body weight	438.5 ± 9.9	402.8 ± 8.3 *	471.7 ± 8.4 *^#^	413.4 ± 7.3 *^$^
Weight gain (%)	39.08 ± 3.6	17.57 ± 5.8 *	36.29 ± 6.2 *^#^	10.89 ± 3.5 *^$^

## Data Availability

The main data are available with the corresponding author.
